# Will coronary artery bypass grafting remain a standard of care for elderly patients with multivessel disease in the contemporary era?

**DOI:** 10.1007/s12471-020-01477-z

**Published:** 2020-07-31

**Authors:** M. Ono, Y. Onuma, P. W. Serruys, J. J. Wykrzykowska

**Affiliations:** 1 Department of Cardiology, Heart Center, Amsterdam University Medical Center, University of Amsterdam, Amsterdam, The Netherlands; 2 Department of Cardiology, National University of Ireland Galway, Galway, Ireland; 3 National Heart and Lung Institute, Imperial College London, London, UK

Advanced age plays an important role in the pathogenesis of atherosclerosis. Therefore, older individuals with coronary artery disease tend to have more complex lesions (e.g. left main coronary artery disease or multivessel disease) than younger individuals. However, many randomised trials have excluded elderly patients based on their age or associated comorbidities, making it unclear what the optimal treatment strategy is for elderly patients with complex coronary artery disease. A higher prevalence of concomitant diseases and increased frailty may make clinicians hesitate to opt for a high-risk invasive strategy, such as cardiac surgery, and instead select a less invasive treatment, because of the potentially higher risk of periprocedural complications.

In this issue of the *Netherlands Heart Journal*, Gimbel et al. report that in their retrospective cohort of patients aged ≥75 years with multivessel disease or left main coronary artery disease, coronary artery bypass grafting (CABG) was associated with significantly lower risks of mortality, acute coronary syndrome, repeat revascularisation and recurrent angina than percutaneous coronary intervention (PCI) [1]. In this trial, the completeness of revascularisation was not independently associated with the outcomes. However, the complete revascularisation rate was relatively low, especially in patients who underwent PCI (29.5%). In the Synergy between PCI with Taxus and Cardiac Surgery (SYNTAX) randomised trial, this rate was 56.7% [2]. The main results are in line with those of past observational studies in which patients undergoing CABG showed lower mortality rates than those undergoing PCI or medical therapy [3, 4].

Nonetheless, potential confounders related to the selection of the revascularisation strategy cannot be excluded in an observational study. For example, in the SYNTAX randomised trial, which compared PCI and CABG in patients with three-vessel disease or left main coronary artery disease, patients who had only one of the two revascularisation options were excluded from the randomisation and were entered into nested registries of either PCI or CABG [2]. Compared with patients in the randomised PCI cohort, those in the PCI registry were on average older (71.2 vs 65.2 years) and had a higher mortality risk at 1 year (7.3% vs 4.4%), whereas those in the CABG registry showed a similar mean age (65.7 vs 65.0 years) and 1‑year mortality risk (3.4% vs 3.5%) as the randomised CABG cohort. This discrepancy between PCI and CABG non-randomised cohorts indicate that more complex patients, such as elderly with multiple comorbidities, tend to undergo PCI rather than CABG, which can introduce biases in observational studies.

**Fig. 1:**
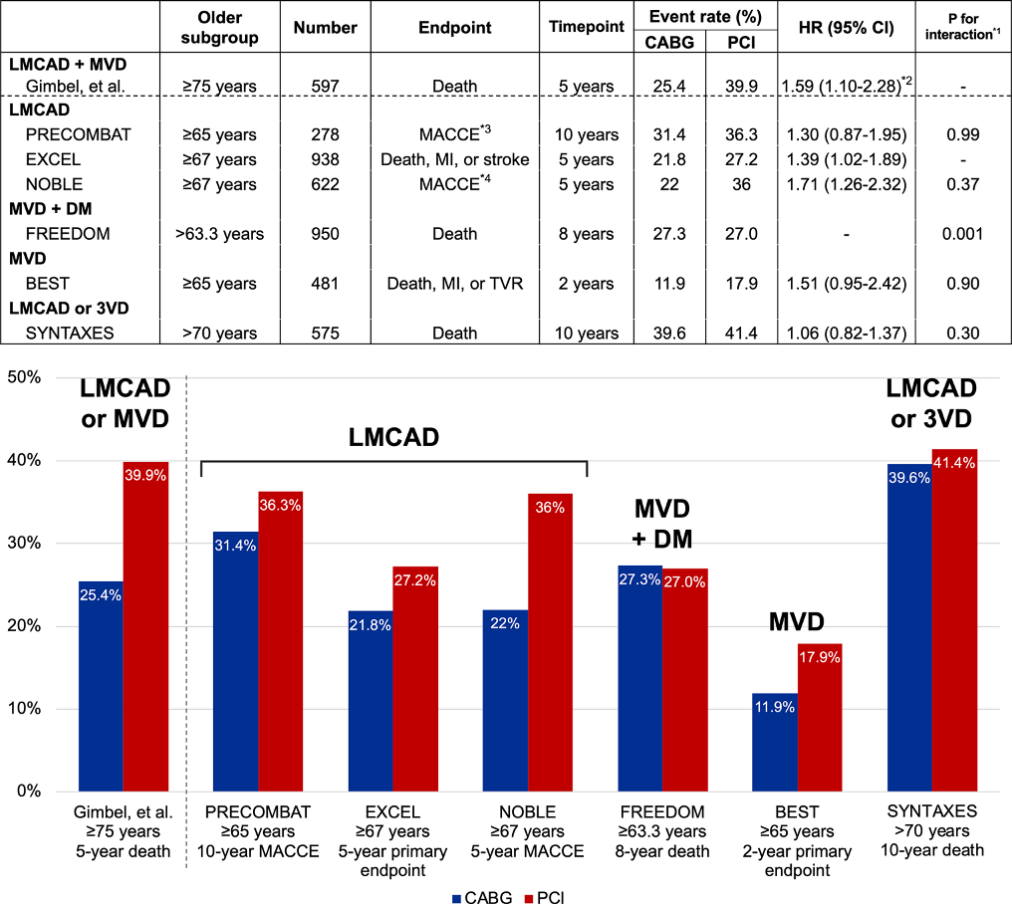
Age-specific subgroups in large randomised trials comparing coronary artery bypass grafting versus percutaneous coronary intervention in patients with multivessel disease or left main coronary artery disease. (^*1^*P* value for interaction represents likelihood of interaction between age-specific subgroups and revascularisation strategy in each randomised trial. ^*2^Adjusted hazard ratio by covariates. ^*3^Defined as a composite of death, MI, stroke and ischaemia-driven TVR. ^*4^Defined as a composite of death, non-procedural MI, repeat revascularisation and stroke. *CABG* coronary artery bypass grafting, *PCI* percutaneous coronary intervention, *HR* hazard ratio, *CI* confidence interval, *LMCAD* left main coronary artery disease, *MVD* multivessel disease, *DM* diabetes mellitus, *3VD* three-vessel disease, *MACCE* major adverse cardiac and cerebrovascular events, *MI* myocardial infarction, *TVR* target vessel revascularisation)

Unfortunately, no randomised trial comparing PCI and CABG specifically has so far been conducted in the older population with left main coronary artery disease or three-vessel disease. Age-specific subgroup analyses of several large randomised controlled trials may provide a less biased evaluation of the impact of the revascularisation strategy on clinical outcomes in those older patients (Fig. 1; [5–9]). However, those studies stratified ‘elderly’ patients by relatively younger age thresholds (63–67 years), and the numbers of elderly patients were limited. Therefore, these results might be underpowered to determine the optimal treatment strategy for elderly patients with complex coronary artery disease. Recently, the results of the SYNTAX Extended Survival (SYNTAXES) trial, which was the extended 10-year follow-up of the SYNTAX trial, were reported. In the SYNTAXES trial, there was no significant difference in the risk of mortality at 10 years between CABG (39.6%) and PCI (41.4%) in the subgroup of patients aged >70 years with three-vessel disease or left main coronary artery disease [10].

Recently, the results of the International Study of Comparative Health Effectiveness with Medical and Invasive Approaches (ISCHEMIA) trial demonstrated that optimal medical therapy alone can be an alternative revascularisation strategy for the management of patients with moderate-to-severe myocardial ischaemia, although no age-specific result has been reported thus far [11]. The strategy of optimal medical therapy, however, does not work effectively without the patient’s compliance and adherence. In the Trial of Invasive versus Medical therapy in the Elderly (TIME) study, optimal medical therapy alone was associated with a higher risk of major adverse cardiac events at 6 months than invasive revascularisation among patients with coronary artery disease >75 years [12]; the compliance and adherence to medical therapy were modest, even in the optimal medical therapy group. Moreover, 45% of patients in the optimal medical therapy group underwent revascularisation during the follow-up because of refractory symptoms.

The adherence to optimal medical therapy is also of paramount importance after PCI. However, an optimal regimen, especially regarding antiplatelet therapy, after PCI may be different for elderly and younger patients, since older patients tend to have a higher bleeding risk as well as a higher ischaemic risk than younger patients [13]. In the Clopidogrel Versus Ticagrelor or Prasugrel in Patients Aged 70 Years or Older With non-ST-elevation Acute Coronary Syndrome (POPular AGE) trial, clopidogrel was preferred over ticagrelor because of a significantly lower bleeding risk without an increase of net clinical events (composite of all-cause death, myocardial infarction, stroke, and bleeding) in patients aged ≥70 years with a clinical presentation of non-ST-elevation acute coronary syndrome [14]. The discrepancy in the results between this elderly-specific study and past studies, such as the Platelet Inhibition and Patient Outcomes (PLATO) trial [15], suggests that the low bleeding risk antiplatelet strategy rather than a potent anti-ischaemic regimen would be favourable in elderly patients in the context of the contemporary PCI era. A recently proposed antiplatelet strategy, namely P2Y_12_ inhibitor monotherapy, has the potential to further improve the prognosis of elderly patients undergoing PCI [16].

As mentioned above, observational studies have an inherent limitation in comparing CABG and PCI. However, those trials suggest that CABG can achieve more favourable outcomes than PCI in a selected elderly population. Dedicated randomised trials comparing CABG versus PCI with a contemporary management strategy in elderly patients are warranted. Until the results of such trials are available, it is important that the Heart Team carefully evaluates the indication for CABG versus PCI versus optimal medical therapy on a case-by-case basis in the elderly population.
